# CAREGIVING AFFECTS WORKING MEMORY, HOPELESSNESS, AND PERCEIVED CONTROL: A MEDIATION MODEL

**DOI:** 10.1093/geroni/igae098.3039

**Published:** 2024-12-31

**Authors:** Valerie Luskey, William Zimbinski, Michael Buxton, Hannah Apostolou, M Lindsey Jacobs

**Affiliations:** University of Alabama, Tuscaloosa, Alabama, United States; University of Alabama, Tuscaloosa, Alabama, United States; University of Alabama, Tuscaloosa, Alabama, United States; University of Alabama, Northport, Alabama, United States; The University of Alabama, Tuscaloosa, Alabama, United States

## Abstract

Caregiver burden can have negative impacts, both on the caregiver and the person receiving care. One of these effects could include more depressive symptoms in the care recipient, which could impact everyday functioning and quality of life. To explore these relationships, data was used from the Health and Retirement Study in the 2014 Core. Participants included people who answered the questions regarding caregiving, perceived control, hopelessness, and completed a working memory task (n=772; female=59.5%, male=40.5%; age=71.14 (SD=11.88); years of education=11.55 (SD=3.53); race/ethnicity were masked due to confidentiality). Participants with Alzheimer’s or Dementia were excluded, and age was used as a covariate. In a mediation analysis, there was an indirect effect of increased hours of caregiving on hopelessness in the care recipient, b=0.10, SE=0.36, p=0.004, which, in turn, lowered working memory in the person being cared for, b=-0.09, SE=0.04, p=0.03. Additionally, increased caregiving led to decreased perceived control, b=-0.08, SE=0.04, p=0.02; however, this did not translate to decreased working memory, b=-0.051, SE=0.04, p=0.21. Apart from these outcomes, increased caregiving had a direct effect on cognition, b=-0.098, t(767)=-2.73, p=0.007, suggesting it influences other mechanisms that decrease working memory not included in the model. A diagnosis of depression could be an explanatory factor that we were not able to include in the model; however, the model indicates a possible area for intervention. If caregivers receive more support and learn skills to promote hope, that could offer positive contributions to increased working memory—thus, translating to overall increased functioning.

